# Prevalence, perceptions and associated factors of health insurance enrollment among older persons in selected cash grant communities in Ghana: a cross-sectional mixed method

**DOI:** 10.1186/s12877-024-05037-7

**Published:** 2024-05-18

**Authors:** Doris Ottie-Boakye, Ayagah Agula Bawah, Naa Dodua Dodoo, J. K. Anarfi

**Affiliations:** 1 School of Public Health, College of Health Sciences, University of Ghana, Box LG 13, Legon, Accra, Ghana; 2 Regional Institute for Population Studies, University of Ghana-Legon, Box LG 96, Accra, Ghana; 3 African Institute for Development Policy (AFIDEP), City Centre, Box 31024, Lilongwe 3, Malawi

**Keywords:** Health insurance enrollment, Older persons, Cash grant communities, Ghana

## Abstract

**Background:**

Universal Health Coverage has been openly recognized in the United Nations health-related Sustainable Development Goals by 2030, though missing under the Millennium Development Goals. Ghana implemented the National Health Insurance Scheme programme in 2004 to improve financial access to healthcare for its citizens. This programme targeting low-income individuals and households includes an Exempt policy for older persons and indigents. Despite population ageing, evidence of the participation and perceptions of older persons in the scheme in cash grant communities is unknown. Hence, this paper examined the prevalence, perceptions and factors associated with health insurance enrollment among older persons in cash grant communities in Ghana.

**Methods:**

Data were from a cross-sectional household survey of 400 older persons(60 + years) and eight FGDs between 2017 and 2018. For the survey, stratified and simple random sampling techniques were utilised in selecting participants. Purposive and stratified sampling techniques were employed in selecting the focus group discussion participants. Data analyses included descriptive, modified Poisson regression approach tested at a *p*-value of 0.05 and thematic analysis. Stata and Atlas-ti software were used in data management and analyses.

**Results:**

The mean age was 73.7 years. 59.3% were females, 56.5% resided in rural communities, while 34.5% had no formal education. Two-thirds were into agriculture. Three-fourth had non-communicable diseases. Health insurance coverage was 60%, and mainly achieved as Exempt by age. Being a female [Adjusted Prevalence Ratio (APR) 1.29, 95%CI:1.00–1.67], having self-rated health status as bad [APR = 1.34, 95%CI:1.09–1.64] and hospital healthcare utilisation [APR = 1.49, 95%CI:1.28–1.75] were positively significantly associated with health insurance enrollment respectively. Occupation in Agriculture reduced insurance enrollment by 20.0%. Cited reasons for poor perceptions of the scheme included technological challenges and unsatisfactory services.

**Conclusion:**

Health insurance enrollment among older persons in cash grant communities is still not universal. Addressing identified challenges and integrating the views of older persons into the programme have positive implications for securing universal health coverage by 2030.

## Background

The issue of universal health coverage is one of the World Health Organisation’s (WHO) General PROGRAMME of Work for 2019–23 strategic priorities to address healthcare financing in meeting the health-related Sustainable Development Goals (SDGs) by 2030 [1, 2]. Universal Health Coverage (UHC) is the core of the health-related SDG 3, and the world is at the mid-point of implementing the 2030 Agenda [3]. Progress on UHC showed improvements in the service coverage index from 45% in 2000 to 67% in 2019 [3]. Despite these global successes, there are challenges for achieving UHC leading to the falling short of set targets [3]. For instance, the lack of concrete operational steps in the majority of countries, in addition to inadequate public financing for health and the increase in out-of-pocket spending on health as a share of total household expenditure, have contributed to lagging in the UHC progress and catastrophic health spending [4, 5].

Global average life expectancy at birth has increased from 66.8% (2000) to 73.3% (2019) [3]. It is estimated that older persons will be more than the number of under nine (9) children worldwide by 2030 [6]. This may result in direct implications for health and sustainable development [6] which may worsen the already stretched existing health systems. The UHC concept is also important to an ageing population, given that the underlying health conditions of this sub-population may be complicated, particularly due to the outbreak of the current global pandemic [7].

Africa is currently experiencing the fastest growth in population [3]. Given the young population base, this may fuel the population of older persons (60 years and over). It is therefore crucial to identify and address sub-population health inequity to achieve universal health coverage. Social health insurance is an extension to social protection and to promote UHC. To improve access to healthcare for its population, countries such as Ghana, Kenya, South Africa and Zimbabwe have espoused health insurance schemes [8–10]. This will improve enrollment and provide financial protection for vulnerable populations like older persons (60 years and older).

Ghana is one of the countries in the West African sub-region apart from Nigeria to have implemented this programme targeting low-income households and individuals almost two (2) decades ago. The country is experiencing a steady rise in the proportion of older persons (60 years and older). This will greatly influence the aim of UHC [11]. The country reviewed its social protection interventions with cash grants in systematically selected communities across the country where older persons and/or their households have been beneficiaries [12, 13]. The country’s cash grant or cash transfer programme, referred to as the Livelihood Empowerment Against Poverty Programme (LEAP) provides cash and health insurance to impoverished households [12, 13]. Through a nationally generated poverty map and rankings, beneficiaries are selected using a cascading technique from the national, regional, district, community, and through to household levels. The LEAP programme is an unconditional cash transfer for persons ≥ 65 years old without productive capacity resulting from poverty, vulnerability and exclusion [12, 13]. Aside from persons ≥ 65 years, the programme covers the severely disabled without productive capacity, orphaned and vulnerable children, and indigent households with pregnant women or mothers with infants. Additional information on the cash grant programme can be obtained elsewhere [12, 13]. Additionally, the Exemption Policy under the NHIS was introduced to ensure that older persons among others have equitable and universal access to quality packages of essential healthcare [12]. To appreciate and give greater priority to covering the health needs of vulnerable groups in Ghana, particularly those 70 years and older, in December 2022, the Government of Ghana launched and activated the “Free Elder Care Policy” embedded in the National Health Insurance Scheme to address the associated health-related challenges this sub-population face [7].

Previous studies have examined the patterns and variations in the prevalence of health insurance enrollment in different populations in many developing countries such as Kenya, Kyrgyzstan, Bangladesh, and Ghana focusing on urban dwellers [14–18] and the general population [19–21]. In Ghana, recent studies on health insurance coverage in older persons have explored the National Health Insurance Scheme (NHIS) enrollment and the frequency and “timing” of health services utilization [22], the correlates of NHIS enrolment among younger, middle-aged and older adults comparison [11], the determinants of National Health Insurance (NHIS) enrolment between younger and older adults [23], and the effect of enrolment within the NHIS on the utilisation of inpatient and outpatient care among older people aged 50 and over [23]. Limited studies have explored health insurance coverage among this sub-population in cash grant communities in Ghana.

Additionally, the health needs of older persons have been lethargic, and a small proportion of this sub-population benefits from such interventions [12, 24]. Different factors have been recognized to have significant effects on participation in UHC among varied populations including older persons [25–29]. Perceptions associated with providers, insurance schemes and the community have been reported to play important roles in decisions to remain enrolled or voluntarily enrolled in insurance schemes [25]. Previous studies have explored evidence of health insurance from different populations [25, 26, 29–31]. Others utilized varied study designs [25, 29–31] and research methodologies [25, 26, 30]. However, the paucity of studies in Ghana focusing on cash grant communities justifies enquiry into the participation, perceptions and associated factors with health insurance enrollment among older persons using a cross-sectional mixed-methods focus of our study. Thus, this study provides a modest contribution to the literature on identified gaps in the prevalence of health insurance enrollment, associated factors and perceptions among older persons in cash grant communities in Ghana’s context.

### Methodology

This study utilized data from the Ageing, Social Protection and Health Systems (ASPHS) study collected in eight (8) cash grant communities in the middle belt of Ghana. The ASPHS data were collected from 2017 to 2018 using a cross-sectional mixed methods research design. The quantitative data used covered 400 non-institutionalized older persons (60 years and above) sampled through stratified (location and sex) and simple random sampling techniques. Structured questionnaires embedded in electronic devices were used for the data collection administered in-person by trained research assistants covering demographic, socioeconomic, behavioural and lifestyle risks, health and health behaviours, and work history. Other information collected covered disability and social protection participation. The estimated burden of time for each questionnaire was about 69 min. Participants for this study were those with complete information on the variables of interest. These included being ≥ 60 years and having information on NHIS enrollment status. Detailed information on the quantitative phase and the selected sites of the study has been documented elsewhere [12, 13].

Further, qualitative data in the form of eight (8) focus group discussions with purposively sampled older persons by different segmentation (sex – men and women and location – rural and urban) from the ASPHS study were utilized in this current research work. A semi-structured focus group discussion (FGD) guide was used for the data collection among 25 men and 35 women. Discussions were in the main local dialect, Asante-Twi, and carried out in locations such as school buildings and church halls. All discussions were audio-taped and, on average lasted for 47 min. Audio tapes from the discussions were transcribed and supported with field notes. The average number of participants for each group discussion was seven (7). Data extracted for this present study was information on older persons’ perceptions of the health insurance scheme.

### Study variables

#### Outcome variable

Health insurance enrollment was measured as a dichotomous variable indicating ‘enrolled’ or ‘not enrolled’ into the national health insurance scheme at the time of the data collection. Study participants were classified as ‘enrolled’ or ‘not enrolled’ based on the verified NHIS card. Participants with valid/active NHIS cards or who self-reported as registered members and could access healthcare based on their registration status were classified as being ‘enrolled’ with health insurance whereas those without valid/active cards or could not report as having been registered and access healthcare based on their status were categorised as ‘not enrolled’ with the health insurance scheme.

#### Explanatory variables

These variables were drawn from demographic, socio-economic, living arrangement, lifestyle risk and health-related factors. A demographic factor like age (60–74 years = 1 as young-old, 75–84 years = 2 as old-old, 85 years or more = 3 as oldest-old) was guided by the international functional age brackets for older persons [12]. Other demographic variables were sex (female = 1, male = 2), marital status (married = 1, not married = 2), and place of residence (rural = 1, urban = 2). Socio-economic variables used were education level attained (no education = 1, primary = 2, middle school = 3, secondary and above = 4), occupation (no occupation = 1, agriculture = 2, non-agriculture = 3), household wealth index (poor = 1, middle = 2, rich = 3), household food security (food secured = 1, not food secured = 2), and cash grant beneficiary status (beneficiary = 1, not beneficiary = 2). The household wealth index was generated based on household living assets and possessions [12, 13, 32]. Household food security was constructed based on participants’ households’ situation about availability and access to food within the last 30 days before data collection [12, 13, 33, 34]. Living arrangements variables were household size (living alone = 1, 2–3 members = 2, 4 or more members = 3), and the presence of a caregiver (no caregiver = 1, living with caregiver in same household = 2, living in a separate household with caregiver = 3). Lifestyle risk variables included the consumption of tobacco (Ever smoked = 1, Never smoked = 2) and the consumption of alcohol (Ever consumed = 1, Never consumed = 2) [12, 13]. Self-rated health status (Bad = 1, Moderate = 2, Good = 3), having non-communicable diseases (NCDs) (Yes = 1, No = 0), and place of seeking care (Non-hospital = 0, Hospital = 1) were the health-related variables.

### Statistical analysis

**Fig. 1:**
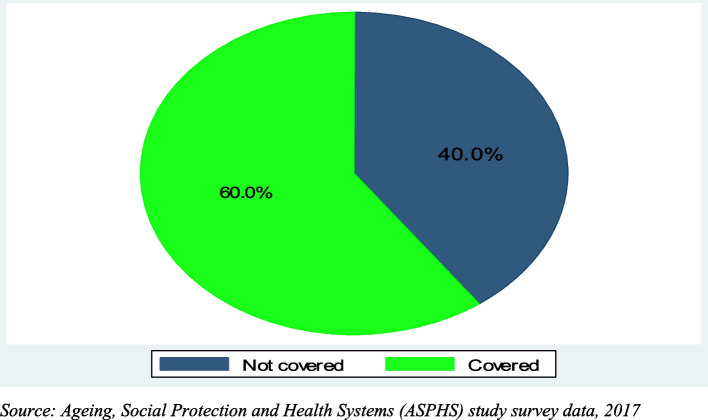
Prevalence of health insurance coverage among older persons (60 + years). Source: Ageing, Social Protection and Health Systems (ASPHS) study survey data, 2017

**Fig. 2:**
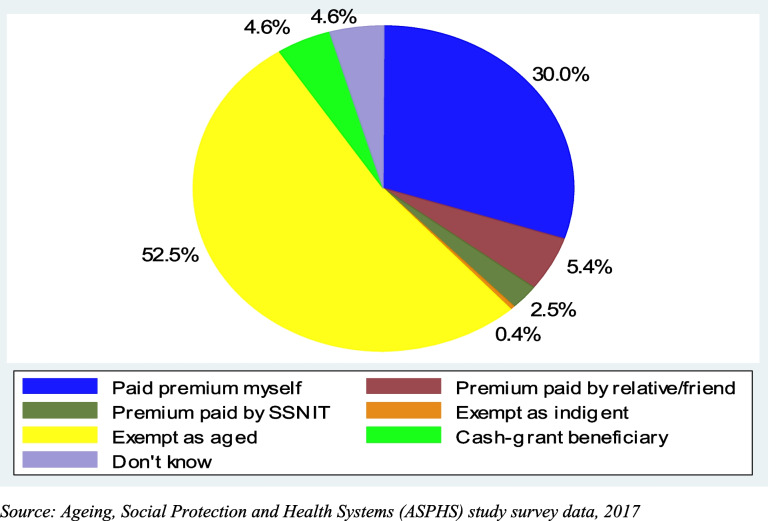
Membership of health insurance coverage achieved among older persons (60 + years). Source: Ageing, Social Protection and Health Systems (ASPHS) study survey data, 2017





We employed descriptive and inferential analytical approaches. First, descriptive analyses were used to describe the background characteristics of the study participants for both the survey and focus group discussions (Tables 1 and 2). Further, this study examined the prevalence of health insurance enrollment and how membership status was achieved (Figs. 1 and 2). Multicollinearity was examined among the variables using the Variance Inflation Factor (VIF). Findings from the VIF of all the variables in this study showed an absence of multicollinearity. Further, the mean VIF was less than 10 (1.37). A modified Poisson regression approach was utilized to examine the variables that were associated with health insurance enrollment. A significant level of 0.05 and an adjusted prevalence ratio (APR) with a 95% confidence interval (CI) were reported. In cross-sectional studies, modified Poisson regression models are suitable when the outcome of interest is not rare [35]. In this study, the modified Poisson regression model used was the Poisson regression of binomial data, and the robust error variance was applied [35]. This regression model is appropriate compared to applying the logistic regression models to binary data [35]. Further details on the Poisson regression model have been explained elsewhere [35]. The equation for the model is given as:

**Table 1:** Background characteristics of survey participants (60 years and older)

**Mean Age (years)**		73.67
	**Percent**	**Number**
***Background Characteristics***		
*Demographic*		
**Age, years**		
Young-old	56.7	227
Old-old	29.3	117
Oldest-old	14.0	56
**Sex**		
Female	59.3	237
Male	40.7	163
**Marital status**		
Married	37.5	150
Not married	62.5	250
**Place of residence**		
Rural	56.5	226
Urban	43.5	174
*Socio-economic*		
**Education level attained**		
No education	34.5	138
Primary	16.8	67
Middle school	39.7	159
Sec +	9.0	36
**Occupation**		
No occupation	41.5	166
Agriculture	42.8	171
Non-agriculture	15.7	63
**Household wealth index**		
Poor	33.2	133
Middle	33.3	133
Rich	33.5	134
**Household food security**		
Food secured	66.5	266
Not food secured	33.5	134
**Cash-grant beneficiary**		
Yes	15.5	64
No	84.5	338
*Living arrangements*		
**Household size**		
Living alone	32.5	130
2–3 members	35.2	141
4 or more members	32.0	128
Don’t know	0.3	1
**Presence of caregiver**		
No caregiver	22.3	89
Same household with caregiver	39.0	156
Separate household with caregiver	38.7	155
*Lifestyle choices*		
**Tobacco use**		
Ever smoked	17.3	69
Never smoked	82.7	331
**Alcohol use**		
Ever consumed	39.7	159
Never consumed	60.3	241
*Health-related factors*		
**Self-rated health status**		
Good	37.0	148
Moderate	35.5	142
Bad	27.5	110
**Presence of at least one chronic illness (NCD)**		
Yes	77.3	309
No	22.7	91
**Place of accessing healthcare**		
Hospital	40.0	160
Non-hospital	60.0	240
**Total**	100.0	400

**Table 2:** Background characteristics of FGD participants (60 years and older)

**Mean Age (years)**		73.67
	**Percent**	**Number**
***Background Characteristics***		
*Demographic*		
**Sex**		
Female	58.3	35
Male	41.7	25
**Marital status**		
Married	40.0	24
Not married	58.3	35
Don’t know	1.7	1
**Place of residence**		
Rural	46.7	28
Urban	53.3	32
*Socio-economic*		
**Education level attained**		
No education	53.3	32
Primary	1.7	1
Middle school	38.3	23
Sec +	5.0	3
Don’t know	1.7	1
**Occupation**		
No occupation	33.3	20
Agriculture	61.7	37
Non-agriculture	3.3	2
Don’t know	1.7	1
**Total**	**100.0**	**400**

Source: Ageing, Social Protection and Health Systems (ASPHS) study FGD data, 2017

where  is the probability of experiencing the outcome of interest for subject i, β’s is the mean of the *ith* subject and approximates relative ratios as exp (β) [34].

All analyses were done with STATA version 14.1.

To explore participants’ perceptions of the health insurance scheme, audio files obtained from the eight (8) FGDs were translated and transcribed into English text for analysis and supported by information from the field notes. Both thematic and content analyses were employed. The study used a semi-structured interview guide to elicit responses on perceptions surrounding the insurance scheme. Probing questions were employed to reach a saturation point with no new perception emerging and to gain more insights. The first author conducted the first steps of the analysis, while all four (4) researchers participated in the determination of the organizing themes and basic themes. The reading and re-reading of transcripts uncovered categories and associated concepts on health insurance aspects examined. Transcripts were read severally to extract participants’ perceptions about the health insurance scheme through the systematic classification process of coding and identifying themes concerning the study’s research questions [36, 37]. Three levels of themes emerged in the data analysis; these were global themes, organizing themes and basic themes. All authors verified generated outputs and discussed them to reach a consensus on the final thematic framework and interpretation. Data were managed and analysed with the help of the Atlas-ti software.

### Ethical considerations

The data used for this study received ethical approval from the Ethics Committee for Humanities (ECH) of the Institute of Statistical, Social and Economic Research (ISSER), University of Ghana-Legon (Protocol Number: ECH 096/16–17), and the Ghana Health Service (GHS) Ethics Review Committee (ERC) (Protocol Number: GHS-ERC: 03/05/17). Permissions were sought from the Regional Health Directorate and the Ministry of Gender, Children and Social Protection/Department of Social Welfare, all in the Ashanti Region. Additionally, the study had permission from the Mampong Municipal Assembly, Health Directorate, Department of Social Welfare, and the National Health Insurance Office at the municipal level respectively. Study participants received detailed information regarding this research. Study participants gave written informed consent to participate in this research work.

### Limitations

This study has some limitations. Firstly, the cross-sectional research design nature of the study does not allow for the cause and effect of findings to be determined. Secondly, findings from the qualitative phase of the study cannot be generalized among older persons residing in cash grant communities and beyond. Thirdly, this study did not explore the individual experiences and institutional factors influencing health insurance enrollment among older persons in cash grant communities. Despite these flaws, utilising a mixed research methodology approach for this study complements these flaws associated with the different research methodologies employed. Hence, this provides a much broader and in-depth overview of the prevalence of health insurance enrollment, its associated factors and perceptions among older persons in cash grant communities in Ghana.

## Results

### Descriptive statistics

#### Background characteristics of study participants

Table 1 presents the background characteristics of the survey participants. The average age was 73.7 years. More than half (56.7%) were in the age group 60–74 years (young-old age). Close to 60.0% were females. About 63.0% were not married. 56.5% resided in rural communities. Some 39.7% had attained a middle school level of education. A two-fifth of the participants had no occupation nor were into agriculture respectively. An equal proportion of one-third of the participants was from poor, middle and rich households respectively. About sixty-seven percent were from households with food security. Eighty-five percent were non-beneficiaries of any cash grant programme. One-third of participants lived alone, and resided in 2–3- and 4 or more-member households respectively. A 22.3% had no form of caregiving. Eighty-three percent had no history of smoking while 60.3% were lifetime abstainers of alcohol consumption. On average, one-third of participants perceived their health status to be good, moderate or bad respectively. A proportion of 77.3% had or suffered from at least one form of NCD such as stroke, hypertension or diabetes. Two-thirds (60.0%) of participants accessed healthcare at the hospital level.

Table 2 presents the background characteristics of the focus group discussion participants. The mean age of the 60 FGD participants was 73.7 years. Close to 60.0% were females (58.3%) and not married (58.3%) respectively. Those who were not married included widowed (43.7%), divorced/separated (18.5%), and never married (0.3%). More than half (53.3%) were urban dwellers and had no formal education respectively. While one-third had no form of occupation, 61.7% were engaged in agriculture.

### Prevalence of health insurance enrollment in cash grant communities in Ghana’s middle belt

Figure 1 presents the prevalence of health insurance enrollment among older persons (60 years and above) in eight cash grant communities included in our analysis. The overall prevalence of health insurance enrollment was 60.0%.

Figure 2 presents how health insurance membership status was achieved among insured study participants. More than half (52.5%) achieved insurance membership by exempt as aged. One-third got covered by the scheme by paying premiums themselves while about five percent (5.4%) had relatives or friends paying for premiums for them to benefit from the scheme.

### Analysis of factors associated with the prevalence of health insurance enrollment among older persons in cash grant communities in Ghana

We present a multi-variable modified Poisson regression model on the factors associated with health insurance enrollment (Table 3). Findings from the full model showed that sex, occupation, self-rated health status, and place of accessing healthcare were associated with health insurance enrollment among older persons in cash grant communities in the middle belt of Ghana. These variables were statistically significant. Whereas occupation was negatively statistically significant with NHIS enrollment, the rest of the significant variables were positive. For sex, this study showed that the likelihood of females [APR = 1.29, 95% CI:1.00–1.67] to be covered by health insurance was 29.0% higher compared to their male counterparts. Regarding occupation, older persons engaged in agriculture were less likely to be enrollees of the scheme relative to those without any form of occupation. Occupation in agriculture reduced enrolment by 20.0% [APR = 0.80, 95% CI: 0.65–0.98]. With self-reported health status, participants who reported having bad health status [APR = 1.34, 95% CI: 1.09–1.64] were 34.0% more likely to be enrolled on the health insurance scheme relative to those with good self-rated health status. For the place of accessing healthcare services, we found that the likelihood of NHIS enrollment among those who accessed healthcare at the hospital level [APR = 1.49, 95% CI: 1.28–1.75] was 49.0% higher compared to those who did not access healthcare at the hospital level.

**Table 3:** A application of a modified Poisson model in identifying predictors associated with health insurance enrollment among older persons in cash-grant communities

Factors	Prevalence risk ratio	*P*-value	Robust
	**[95%CI]**		**Standard error**
*Demographic*			
**Age, years**			
Young-old [59–73] [RC]	1		
Old-old [75–84]	1.13[0.94–1.35]	0.181	0.104
Oldest-old [85 +]	0.80[0.60–1.07]	0.133	0.116
**Sex**			
Male [RC]	1		
Female	1.29 [1.00–1.67]	0.049*	0.169
**Marital status**			
Not married [RC]	1		
Married	1.20 [0.95–1.53]	0.417	0.078
**Place of residence**			
Rural [RC]	1		
Urban	0.93 [0.79–1.10]	0.417	0.078
*Socio-economic*			
**Education level attained**			
No education [RC]	1		
Primary	0.83 [0.64–1.06]	0.139	0.106
Middle school	0.91 [0.75–1.11]	0.347	0.090
Sec +	0.75 [0.52–1.07]	0.117	0.138
**Occupation**			
No occupation [RC]	1		
Agriculture	0.80 [0.65–0.98]	0.032*	0.083
Non-agriculture	1.21 [0.99–1.48]	0.060	0.125
**Household wealth index**			
Poor [RC]	1		
Middle	0.91 [0.75–1.10]	0.032*	0.083
Rich	1.01 [0.83–1.23]	0.060	0.125
**Household food security**			
Not food secured [RC]	1		
Food secured	1.17 [0.98–1.40]	0.077	0.107
**Cash-grant beneficiary**			
Yes [RC]	1		
No	0.85 [0.69–1.04]	0.124	0.089
*Living arrangements*			
**Household size**			
Living alone [RC]	1		
2–3 members	1.04 [0.86–1.27]	0.658	0.105
4 or more members	0.86 [0.69–1.08]	0.199	0.099
**Presence of caregiver**			
No caregiver	0.99 [0.78–1.26]	0.949	0.121
Same household	1.05 [0.86–1.28]	0.633	0.107
Separate household [RC]	1		
*Lifestyle choices*			
**Tobacco use**			
Never smoked[RC]	1		
Ever smoked	1.14 [0.88–1.47]	0.313	0.147
**Alcohol use**			
Ever consumed [RC]	1		
Never consumed	1.05 [0.88–1.25]	0.591	0.094
*Health-related factors*			
**Self-rated health status**			
Good [RC]	1		
Moderate	1.21 [0.98–1.50]	0.071	0.130
Bad	1.34 [1.09–1.64]	0.005*	0.139
**Presence of at least one chronic illness (NCD)**			
Yes [RC]	1		
No	0.90 [0.71–1.15]	0.418	0.112
**Place of accessing healthcare**			
Non-hospital [RC]	1		
Hospital	1.49 [1.28–1.75]	0.000*	0.120

*RC* Reference Category

Source: Ageing, Social Protection and Health Systems (ASPHS) study survey data, 2017

^*^p < 0.05

### Study participants’ perceptions about the National Health Insurance Scheme

**Fig. 3:**
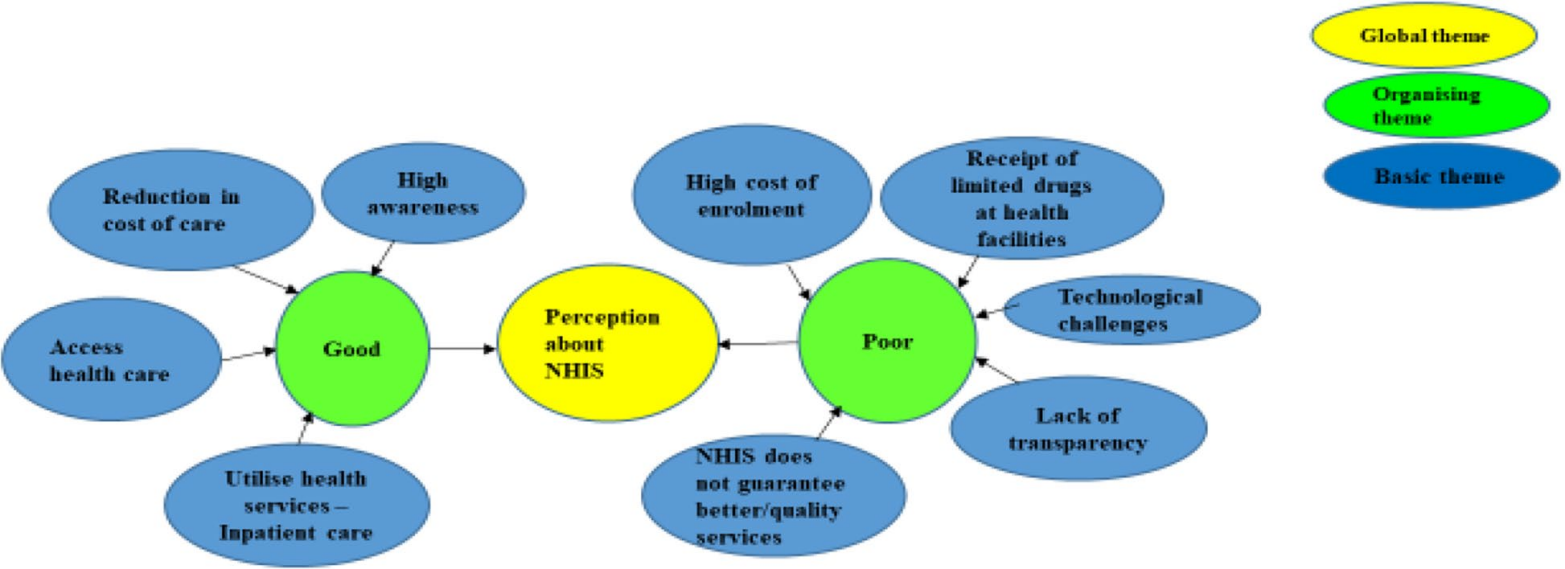
Perceptions about health insurance emergent global theme, organized themes and basic themes

The perceptions of participants about the scheme were varied and wide-ranging in themes. Two (2) broad themes emerged. These were “good” and “poor” perceptions shown in the networking Fig. 3. Four (4) and five (5) sub-themes further emerged from the two broad themes, “good” and “poor” respectively (Fig. 3).

### Good perceptions about the health insurance scheme

Regarding good perceptions about NHIS, the sub-themes included high awareness, reduction in cost of care, access to healthcare, and the utilization of in-patient care services.

#### High awareness

On the high awareness of the insurance scheme, participants revealed that the older person has a high awareness of the existence and benefits of this form of social protection. To participants, its discussion on the traditional media, particularly, on the radio has made it widely known. Other participants acknowledged the use of community announcements and visitation by insurance officers on the need to get enrolled on the scheme. This was pointed out by female FGD participants in both rural and urban areas. The following quotes illustrate two female FGD participants’ explanations of the high awareness of insurance schemes with a focus on the sources of receiving information.

“It is widely known. Everyone is aware of the insurance. It is announced and discussed usually on the radio” – (Women, Rural FGD).

“It is usually announced that everyone goes and registers for the NHIS. Sometimes the officers come to the community to do the announcement and we usually go to the Municipal capital to do the registration” – (Women, Urban FGD).

#### Reduction in cost of healthcare

To study participants, health insurance coverage reduces the cost of healthcare. At the point of seeking care, insurers compared to non-insurers have to spend less. Participants appreciated the existence and importance of the health insurance scheme. Some female participants in rural FGDs remarked:

“If one compares herself to how much those with no NHIS card spend as against you who has the card at the facility, then one has no other option than to appreciate the existence and the importance of the NHIS programme”—(Women, Rural FGD).

“…with no insurance, you are made to pay for every service you are given.” – (Women, Rural FGD).

#### Access to healthcare

Access to healthcare was one of the major themes. While financial access to care was mentioned, others emphasized geographic accessibility, that is, an opportunity to visit any health facility of their choice irrespective of one’s location given the use of the insurance card. A couple of responses from FGD participants below illustrate this sub-theme:

“…once you have the card, you can access healthcare.” – (Men, Urban FGD).

“One could go and seek care anywhere of her choice.” – (Women, Urban FGD).

“Yes. We can access healthcare from any facility.” – (Men, Rural FGD)

One participant recounted her experience:

“I did my insurance at District A and I have been able to access healthcare in Municipality B since I came here.” – (Women, Urban FGD).

#### Utilisation of in-patient care

A few participants cited the utilisation of in-patient care services as one of the good perceptions of the insurance scheme. Participants expressed how beneficial health insurance coverage becomes at the point of hospitalisation or on admission. The next quote illustrates utilising in-patient care service sub-theme:

“It’s been very helpful especially when you go on admission with it. …, you do not pay for any other cost when on admission” – (Women, Rural FGD).

#### Poor perceptions about health insurance scheme

Participants expressed poor perceptions about health insurance too. Per their observations, five (5) sub-themes emerged (Fig. 3): high cost of enrolment, technological challenges, receipt of limited drugs at health facilities, lack of transparency, and limited guarantee for quality health services.

#### High cost of enrollment

As for the high cost of enrollment, some participants recounted the high cost of premiums in general and the inequity in the premium payment among older persons. The following quotes illustrate the themes:

“Those older persons who are 70 years and above do pay GH¢ 8.00. Those of us who are between the ages of 60 and 70 years, do pay GH¢ 26.00. Older persons 70 years and above pay the same amount of premium as children. For renewal, the older ones pay GH¢ 5.00. Those of us who are not yet 70 years old, we pay GH¢ 23.00 for renewal.” - (Women, Urban FGD).

“The premium cost is too high. They should consider GH¢ 10.00 instead of the GH¢ 26.00 that we pay. The premium cost of GH¢ 26.00 is too expensive.” - (Women, Urban FGD).

#### Receipt of limited drugs at health facilities

Receipt of limited drugs at health facilities was also stated by some participants. At the point of seeking care, insurers receive limited required drugs or have to purchase drugs elsewhere, and in some cases, drugs offered are not meant for the exact illness. Regarding limited drugs at health facilities, some participants remarked:

“Other times too, the prescription form is given to you to make the purchase yourself but if you have to receive any medication, you will be given Paracetamol and B-complex.” – (Men, Rural FGD).

“Sometimes the drugs prescribed and given are not even meant for the exact illness you came to the health facility to report on!” - (Women, Urban FGD).

“We have insurance. But, we are asked to pay money when seeking care” – (Women, FGD Rural).

#### Technological challenges

Technological challenges such as poor internet connectivity associated with the health insurance scheme were raised by participants. Such occurrences are sometimes the opportunity for officers to extort monies from potential clients. To this effect, some participants indicated:

“Usually, you are told the network (internet) is not working. …Though, the challenge with the network (system down problem) is always cited as the reason, yet, the moment one offers extra money, the so-called network problem will cease” - (Women, Urban FGD).

“The poor internet connection has been a major setback for us in obtaining our NHIS cards on time” – (Women, Rural FGD).

#### Lack of transparency

Concerning the lack of transparency about getting an insurance card, participants bemoaned the delay in receipt of the card, in particular, new enrollees. Others cited the travel time to the district capital to access such services and the challenge of associated transportation costs. Others highlighted the health services that come with using the card. For example, some participants said:

“There is no transparency about getting the insurance card. Whether a new enrollee or renewing the card, one will sometimes spend about three days continuously at the office. And because we have to travel to the municipal capital to get to the office of the NHIS, sometimes transportation cost also becomes a challenge.” – (Men, Rural FGD)

“The issue is that there is no transparency because aside from presenting this card, we are expected to pay for the services rendered to us. It is because of the monies they take from us at the health facility which we see as extortions, which we are complaining about.” – (Men, Urban FGD).

#### No guarantee of quality healthcare

Participants perceived that health insurance coverage does not guarantee quality healthcare at the point of service utilization. Some participants stated emphatically that quality healthcare would be realized when users are ready to pay for it. Others complained and equated quality healthcare to the limited number of drugs received at the point of accessing healthcare. This sub-theme is illustrated by the statements below:

“If one has the money to pay for the services, then you will have quality healthcare. If not, with the NHIS only, you will be given paracetamol as your medication” – (Women, Urban-FGD).

“Unless you are ready to pay. Once you are ready to pay, you will receive quality Healthcare.” – (Women, Rural FGD).

“The use of the NHIS does not promote quality healthcare since the number of prescribed drugs is not enough” – (Women, Urban FGD).

## Discussion

This study sought to examine (1) the prevalence of health insurance enrollment, (2) the factors associated with health insurance enrollment among older persons in cash grant communities, and (3) older persons’ perceptions of the health insurance scheme. In this section, the research work will discuss the major findings.

We found the overall prevalence of health insurance enrollment among older persons in cash grant communities to be 60.0%. Despite Ghana’s implemented policies to promote universal health coverage for all ages, older persons in cash grant communities are yet to experience universal access to healthcare. In other jurisdictions such as the Philippines, coverage among older adults rose from 9.4% (2003) to 87.6% (2017) after the introduction of the mandatory National Health Insurance Programme (NHIP). Ghana’s health insurance coverage has seen fluctuations between 2014 (39.0%) and 2018 (36.0%), and translated into active membership of 10.55 million (2014) and 10.66 million (2018) [7]. Earlier studies utilizing nationally representative data among older persons [70 + years] showed 43.0% coverage under the old-age exemption policy [30]. Though more than half achieved their health insurance membership by the current Exempt policy under the NHIS, one in every three paid health insurance premiums by self. Using nationally representative data, the authors found that more than half of older persons (70 years and older) paid premiums for their health insurance membership despite the exemption [30]. Corroborating findings from this current study, [30] found that a little above one-third of older adults fund their health insurance premiums by themselves. Evidence shows that older persons in many countries in Sub-Saharan Africa do not benefit from health insurance coverage [23, 38, 39]. This has been attributed to the limited or non-existence of policies targeting older persons’ access to healthcare [30]. The gap in health insurance coverage among older persons has implications for accessing healthcare. In a similar district in the region of the present study area, [31] reported that older persons finance their healthcare utilization through personal income despite the introduction of health insurance, particularly, among poor older persons.

Further, this study observed that the sex of participants positively affected health insurance enrollment. Exploring health insurance coverage by sex from the data for this study, it showed that females are 15.3 percentage points higher than male counterparts to be covered by health insurance. Females have been reported to be higher users of healthcare among poor older persons in Ghana [31]. The authors attributed it to the country’s cash grant programme, Livelihood Empowerment Against Poverty (LEAP). Findings from this present study also revealed that one (1) in every 20 of the study participants achieved health insurance membership through this cash grant programme. Contrary to this study’s findings, earlier studies have reported higher coverage among males than females [40–43].

The findings from this study highlight the importance of occupation in health insurance enrollment in cash grant communities in Ghana. Three in every five older persons are engaged in agriculture among both the survey and FGD participants. In this study, participants are more likely to reside in rural areas, and similar studies have supported this finding [12, 13, 44, 45]. This could be attributed to the fact that persons engaged in agriculture are more likely to be found in the informal sector, and rural areas, and may largely be non-contributors to social security which guarantees automatic health insurance coverage. This may also account for the low (2.5%) achievement of health insurance membership through social security among participants in this study [13] averred that older persons engaged in agriculture operate on small scale bases, and have limited access to income and social security. Corroborating findings from this current study from three national household surveys, [46] found the association between occupation and health insurance coverage in Ghana. Those working in the professional sector, skilled-manual, sales and even the unemployed were more likely to be health insurance enrollees compared to those in the agriculture sector [46]. The authors attributed this inequity to the lack of reliable income among workers in the agricultural sector. Other studies have attributed it to the rurality of agriculture engagements, and may often reside in rural settings where transport costs for insurance enrollment may be a barrier [47]). Nevertheless, there is evidence of a positive association between employment and health insurance coverage [48, 49] reported of the gender dynamics of the correlation between agriculture and health insurance coverage.

Our study discovered that self-rated health status was associated with health insurance enrollment. In other words, older persons with self-rated moderate or bad health status were NHIS enrollees than those with good health status. This may be due to the need for healthcare, and hence, the taking advantage of the financial access to healthcare that national health insurance coverage has been found to guarantee [50, 51]. In this current study, 7 in every 10 reported having had or ever suffered from chronic illness such as stroke, hypertension or diabetes. Hence, a need to utilise more health services. In developed countries such as Ireland, health status was reported to be associated with the purchase of private health insurance [52]. Corroborating this present study, previous studies discovered that persons with poorer health were more likely to be health insurance enrollees due to the need for more healthcare services [52–54], and hence, frequent visits to the hospital [55, 56].

The study further observed that accessing healthcare at the hospital was associated with health insurance enrollment. A sizable proportion (77.3%) of study participants had at least one chronic illness. Health services for such conditions are often rendered at health facilities with hospital status in terms of the required equipment and personnel and are likely to be NHIS accredited. The National Health Insurance Scheme besides the premium exemption policy for older persons, also provides a benefits package covering in-patient hospital care and out-patient care at primary and secondary levels [57, 58]. Hence, health insurance enrollment provides financial access to such services at the hospital level.

Study participants had varied perceptions about the health insurance scheme. This has implications for their enrollment and continuous participation in the scheme. High awareness of the scheme and benefits, reduction in the cost of care, access to healthcare, and the utilisation of in-patient care were good perceptions about the insurance scheme. Some studies have cited the creation of more awareness of the scheme’s benefits to improve its enrolments [19, 25]. On the reduction of cost of care, earlier studies have supported such observations of enhancing access to health services at a reduced or no cost [23, 59], and the removal or diminished cost burden in service utilisation [23, 60, 61]. It is extensively recognized that Ghana’s National Health Insurance Scheme has improved access to healthcare for many people including older persons despite issues of equity and sustainability [19, 25]. Earlier studies have also highlighted the scheme’s improvement in access to a continuum of care [19, 62]. The effect of health insurance enrollment on service utilisation has widely been documented in different populations in West Africa, China and the United States of America (USA) in the literature [25, 63–66]. For instance, [27] found an increase in the utilisation of in-patient and outpatient care by 6.0% and 9.0% respectively among older adults’ scheme subscribers relative to non-subscribers in Ghana. Other studies have also documented the positive association between health insurance and healthcare utilisation [25, 63–66].

Participants’ poor perceptions of the scheme included the high cost of enrollment, technological challenges, lack of transparency and no guarantee of quality healthcare. These certainly could impede the rapid achievement of universal health coverage. Two in every five of participants in this current study were non-enrollees of the health insurance scheme. Studies among different sub-populations such as informal sector workers in Indonesia found those who experienced financial hardships to be 7.7 percentage points less likely to pay insurance premiums routinely [67]. On technological challenges, existing evidence in the literature shows the internet challenges as one of the operational barriers to health insurance coverage among older adults in Ghana [68, 69]. The need for effective measures to address this challenge to promote UHC, particularly among older persons is a catalyst for achieving SDG 3. One such effective measure proposed has been harmonizing the different ages associated with the various social protection initiatives among older persons in Ghana [12, 69]. For instance, older persons 65 years and above may qualify for cash grants, and pension schemes for those 60 years and above while the exempt policy under the health insurance scheme is for those 70 years and over. Previous studies have reported poor accountability and transparency issues under the scheme [70, 71]. Participants’ perception of non-guaranteed quality healthcare is a threat to the scheme’s effectiveness and sustainability [72]. Nketiah-Amponsah et. al., reported the geographic variations in quality healthcare under the scheme using nationally representative survey data. Enrollees in rural settings cited a better perception of the quality of services compared to urban enrollees. Other researchers have reported otherwise. For instance, in older adults in Ghana 88.8% perceived treatment received as good, and 90.6% ranked the quality of healthcare as good [59].

## Conclusion

Despite the progress made by Ghana in policy formulation on achieving universal health coverage, especially, for older persons, there remains a coverage gap with the implementation of these policies. This study highlights the fact that health insurance enrollment among older persons in cash grant communities where the most vulnerable populations are perceived to reside is not universal, and is characterized by inequities and mixed perceptions. Addressing challenges associated with this social insurance intervention targeting low-income households and individuals while incorporating its strengths highlighted will put this sub-population in cash grant-targeted communities on track to securing UHC by 2030 and promoting inclusivity and responsiveness.

## Recommendations and policy implications

Future mixed methods could explore older persons’ lived experiences and institutional factors to understand how the various social protection programmes ensure health insurance enrollment and financial protection for older populations, especially in cash grant communities. Deliberate efforts are needed to consciously prioritize older persons in social protection programmes to ensure full participation and benefits, particularly in cash grant communities in promoting UHC in the quest to achieve SDG 3 by 2030.

For policy implications, our findings suggest the need for governments and key stakeholders to make this sub-population a centre of interest in their innovative strategies and health insurance policy reforms despite of existing frameworks/policies to achieve complete coverage specifically in cash grant communities. Additionally, our results stress the importance of a road map to strengthen cash grant communities and relevant stakeholders’ engagements on the scheme’s services, programmes, activities and entitlements particularly concerning older persons through information, education and communication. Finally, promoting and ensuring sustained non-contributory universal social security for old age and ageing would be an effective way of promoting old age security, and ultimately increase the well-being of older persons, especially, in cash grant communities.

## Data Availability

The datasets used and/or analysed during the current study are available from the corresponding author on reasonable request.

## Abbreviations

APR: Adjusted Prevalence Ratio
ASPHS: Ageing, Social Protection and Health Systems
CI: Confidence Interval
ECH: Ethics Committee for Humanities
ERC: Ethics Review Committee
FGDs: Focus Group Discussions
GHS: Ghana Health Service
GSS: Ghana Statistical Service
ISSER: Institute of Statistical, Social and Economic Research
LEAP: Livelihood Empowerment Against Poverty
NCDs: Non-Communicable Diseases
NHIA: National Health Insurance Authority
NHIP: National Health Insurance Programme
NHIS: National Health Insurance Scheme
SDGs: Sustainable Development Goals
UHC: Universal Health Coverage
WB: World Bank
WHO: World Health Organisation
